# Tourniquet-induced nerve compression injuries are caused by high pressure levels and gradients – a review of the evidence to guide safe surgical, pre-hospital and blood flow restriction usage

**DOI:** 10.1186/s42490-020-00041-5

**Published:** 2020-05-28

**Authors:** Bassam A. Masri, Andrew Eisen, Clive P. Duncan, James A. McEwen

**Affiliations:** 1 Department of Orthopaedics, Faculty of Medicine, University of British Columbia, 207-1099 West 8th Avenue, Vancouver, BC V6H 1C3 Canada; 2 Division of Neurology, Faculty of Medicine, University of British Columbia, Vancouver, Canada; 3 Department of Electrical and Computer Engineering, University of British Columbia, Vancouver, Canada

**Keywords:** Tourniquet, Pressure, Nerve, Compression, Injuries, Safety, Personalization

## Abstract

Tourniquets in orthopaedic surgery safely provide blood free surgical fields, but their use is not without risk. Tourniquets can result in temporary or permanent injury to underlying nerves, muscles, blood vessels and soft tissues. Advances in safety, accuracy and reliability of surgical tourniquet systems have reduced nerve-related injuries by reducing pressure levels and pressure gradients, but that may have resulted in reduced awareness of potential injury mechanisms. Short-term use of pre-hospital tourniquets is effective in preventing life-threatening blood loss, but a better understanding of the differences between tourniquets designed for pre-hospital vs surgical use will provide a framework around which to develop guidelines for admitting to hospital individuals with pre-applied tourniquets. Recent evidence supports the application of tourniquets for blood flow restriction (BFR) therapy to reduce muscular atrophy, increase muscle strength, and stimulate bone growth. BFR therapy when appropriately prescribed can augment a surgeon’s treatment plan, improving patient outcomes and reducing recovery time. Key risks, hazards, and mechanisms of injury for surgical, BFR therapy, and pre-hospital tourniquet use are identified, and a description is given of how advances in personalized tourniquet systems have reduced tourniquet-related injuries in these broader settings, increasing patient safety and how these advances are improving treatment outcomes.

## Background

Tourniquets have been used for centuries to occlude arterial flow distal to the device to control extremity bleeding and provide a clearer surgical field. Generations of tourniquet technology have evolved over time, from simple cloth bands tied tightly around limbs, to mechanical screw tourniquets, to elastic, non-pneumatic Esmarch tourniquets, and most recently to the current generation of microprocessor controlled pneumatic tourniquets first conceived and developed by McEwen in 1981 [1–3]. Reports of limb paralysis, serious nerve damage, and a range of other injuries were common with early generations of tourniquets [2, 4]. However, progressions in recent generations of pneumatic tourniquet technology, highlighted by advances in personalized tourniquets, have substantially reduced the frequency and severity of such injuries [5]. An exception to this progression in safety was evident from the introduction of a non-pneumatic elastic tourniquet ring which produced high pressure gradients and uncontrolled applied pressure levels, resulting in reports of nerve injuries and pulmonary embolism [6–8].

While automatically controlled pneumatic tourniquets have improved the safety, accuracy and reliability of restricting and occluding blood flow into limbs, it remains important to review the evidence of how and why tourniquet-related nerve injuries occur to identify and ameliorate key risk factors associated with tourniquet use. It is estimated that tourniquet systems are used in more than 15,000 surgical procedures around the world daily [8], but their widespread adoption is not without clinical risk of injuries. Skin, muscle, nerve, blood vessels and connective tissue (in isolation or combination) are subject to potential tourniquet damage manifested by numbness, paresis or paralysis with muscle atrophy [2, 8]. Present evidence has demonstrated that lower tourniquet pressure levels and pressure gradients applied for short durations of time decreases both frequency and severity of tourniquet-induced injuries [1, 9]. Nevertheless, clinical protocols for tourniquet usage have not significantly evolved since safer automated technology was introduced [1, 5, 8].

Further motivation to review the existing evidence of the causes of tourniquet-related injuries has arisen from the recent use of tourniquets more broadly in perioperative and non-operative applications, including pre-hospital trauma settings and newly established blood flow restriction (BFR) therapies [1, 3, 10, 11]. The lessons learned through advancements in surgical tourniquet technology should be applied to these broader settings. A better understanding of how tourniquets are being used outside of the surgical setting may enable surgeons to improve the treatment of their patients. Due to key differences in both the design and application of pre-hospital tourniquets compared with classical surgical tourniquets, there is a need for guidelines to establish best practices for admitting patients to the hospital with a pre-applied tourniquet. In another application, the use of surgical-grade tourniquets in blood flow restriction therapy can be incorporated into the surgical treatment plan during both the prehabiliation and rehabilitation phases. BFR therapy has been shown to increase muscle strength and mass at low loads to protect the healing limb, reducing overall recovery times [10]. Surgeons can play a key role in the prescription of safe and effective use of BFR therapy based on their knowledge of risks associated with surgical tourniquet use.

## Main text

### Nerve compression injury

**Fig. 1:**
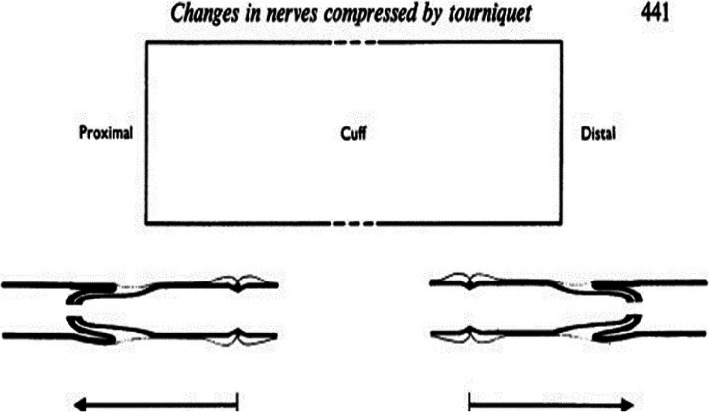
Diagram illustrating the direction of displacement of the nodes of Ranvier due to pressure gradients near the edges of the cuff, i.e. from a high pressure region beneath the middle of the cuff to regions of lower pressure near the edges. (Republished with permission of John Wiley & Sons - Books., from Ochoa J, Fowler TJ, Gilliatt RW. Anatomical changes in peripheral nerves compressed by a pneumatic tourniquet. J Anat 1972;113(Pt 3):433–55; permission conveyed through Copyright Clearance Center, Inc.)

It is almost 50 years since Ochoa et al. [12] demonstrated that the damage to nerve fibers resulting from a compression tourniquet was the direct result of the applied pressure, and not a consequence of secondary ischemia, and that the pressure gradient was higher near the edges rather than in the middle of a tourniquet. Subsequent studies have shown that ischemia is also of relevance, particularly at the distal cuff edge and below [13]. In patients with tourniquet-induced paralysis there is focal conduction delay at the level of the tourniquet border zone which likely reflects the functional equivalent of the structural abnormalities observed by Ochoa et al. (Fig. 1) [12]. Tourniquet compression nerve injuries are typically transient, and resulting symptomatology is mild to moderate [14]. However, when the applied pressure gradient is high, there is a risk of axonal injury with subsequent axonal degeneration and accompanying target muscle fibre atrophy [14]. If this happens recovery may be prolonged (lasting weeks or even months). Further, because of potential mal-innervation, recovery may be incomplete with functionally impaired fractionation of movement and/or permanent sensory deficit. This is especially true when the upper limb is involved. Because upper extremity nerves are closer to the skin surface, or adjacent to bone they are more susceptible to direct compression injury [1, 4]. Upper extremity nerve injuries are more commonly reported than in the lower limb, with the radial nerve being the most vulnerable [1, 15, 16]. Thus location of tourniquet placement is also significant for reducing the risk of direct nerve compression [1, 8, 15].

**Table 1:** Key Insights and Takeaways

Key Insights	
Nerve Injuries	• Transient and permanent nerve injuries can be associated with tourniquet use when not used appropriately.
Pressure Levels and Gradients	• High pressure gradients resulting from narrow cuffs and high applied pressures will cause injury • The lowest effective pressures should be applied, using wide cuffs that conform well to the limb shapes of individual patients
Tourniquet Applications	• For surgical applications, tourniquet pressure levels and tourniquet pressure gradients should be minimized to reduce tourniquet-induced nerve compression injuries. • For pre-hospital applications, there is a need for guidelines on safe tourniquet selection and patient application, and on safe transfer of patients to surgical settings. • For BFR applications, evidence arising from surgical tourniquet development, research and clinical studies should be used to reduce the potential for tourniquet-induced nerve compression injuries.

Although there has been considerable investigation into the pathological sequelae of chronic nerve compression, both in humans and a variety of animal models, much less is known about acute effects of compression as they may occur initially with tourniquet use. In the acute phase of nerve compression conventional nerve conduction studies are of limited value and usually normal. Nerve excitability studies have emerged as a recent novel, non-invasive technique, that allows for the assessment of peripheral axonal biophysical properties that include ion channels, energy-dependent pumps and membrane potential in both health and disease [17, 18]. These biophysical properties likely become deranged during compression.

Temporary compression-related symptoms are common and predominantly sensory. After 30–60 min, inflation of a tourniquet is frequently followed by the development of a dull aching pain, despite adequate regional anaesthesia. Tourniquet pain reflects selective pain transmission of unmyelinated, slowly conducting C fibres, which are continuously stimulated by skin compression.

### Muscle injury

After tourniquet application there is progressive cellular hypoxia, acidosis, and cooling in the occluded limb [19]. This progressively decreases tissue pH and pO2, and increases pCO2, K+, and lactate [19, 20]. Muscle is susceptible to these metabolic changes, and histological evidence of muscle damage is evident 30–60 min after tourniquet inflation [21]. These changes are generally mild and well tolerated. There is increased microvascular permeability with reperfusion, resulting in swelling and tissue oedema, which in some patients can cause a “post-tourniquet syndrome” especially if inflation time has been prolonged. Post-tourniquet syndrome is characterized by a swollen, stiff, pale limb with weakness developing 1–6 weeks after the tourniquet application. High tourniquet pressure levels and applied pressure gradients combined with ischemia may induce more profound damage to muscle than ischemia alone [10, 19].

### Tourniquet pressure gradients

#### Reducing tourniquet pressure

In order to ensure a bloodless field, the application of early-generation tourniquet devices applied unnecessarily high pressure levels to the underlying tissues, with corresponding high applied pressure gradients [2, 6]. Considerable evidence in early literature testifies to the common occurrence of nerve injuries after the use of these tourniquets [1, 2, 4, 6, 16, 22, 23]. Eckhoff (1931) analyzed the causes of tourniquet paralysis, with pleas to use more moderate and controlled applied pressure levels [4]. In a case study Moldaver (1954) described the subtle differences in clinical symptoms associated with tourniquet paralysis, concluding that nerve injury resulted from mechanical pressure localized to a very narrow area [22]. Aho et al. (1983) reported a case of temporary paralysis induced by a pneumatic tourniquet, even though the pressure level was inflated to 250 mmHg for a duration of 75 min. While these parameters are typically considered safe, further investigation revealed the gauge was faulty resulting in application of a much higher than intended pressure level [16]. Several early papers reported that use of elastic bandages and faulty gauges in early, non-microprocessor controlled pneumatic tourniquet systems resulted in the application of high pressure levels and high pressure gradients, and accounted for the majority of reported tourniquet related injuries [2, 16].

Historically, standard pressure levels of 250 mmHg for the upper extremity and 350 mmHg for the lower extremity have been applied [1, 24, 25]. However, these “standard” pressure levels do not take into account patient-specific variables such as: limb shape and circumference, composition of tissue underlying the cuff, vessel compressibility, limb position, systolic blood pressure, and tourniquet cuff application and design [25], often translating into application of higher than necessary pressure levels with increased risk of tissue and nerve injury [8, 9, 24, 26–28]. The introduction of the microprocessor controlled pneumatic tourniquet system was instrumental to the use of lower, less hazardous pressure levels, with the added ability to actively regulate and maintain a set pressure level, thus helping to minimize unanticipated variations [2, 5, 22, 24].

Limb occlusion pressure (LOP) is defined as the minimum pressure required, at a specific time by a specific tourniquet cuff applied to a specific patient’s limb at a specific location, to stop the flow of arterial blood into the limb distal to the cuff [8]. A personalized tourniquet pressure level can be set based on this measured LOP to account for the variables summarized above, plus an additional margin of safety to account for intraoperative changes in anesthesia and patient physiology [1, 5, 24]. The importance of setting tourniquet pressure based on LOP was recently illustrated by Saw and Hee [23], who reported that a patient who had a tourniquet cuff applied to the lower limb at a pressure level of 250 mmHg for 112 min developed a sensory deficit in the distribution of the common peroneal nerve [23]. Despite being below the “standard” pressure level for a lower limb, Saw and Hee concluded that the tourniquet pressure level was likely unnecessarily high and recommended that the pressure level should be personalized using LOP as a guide [23].

Younger et al. [29] importantly observed that by using a tourniquet system to automatically measure individual LOPs, average thigh tourniquet pressures were reduced between 19 and 42% below the typical standard pressures while continuing to maintain an acceptable bloodless field [29]. A lower applied personalized tourniquet pressure level is effective and decreases the applied pressure gradient in the tissue underneath the cuff, reducing the probability of tourniquet-related injuries [1, 5, 8, 24].

#### The importance of tourniquet cuff design

**Fig. 2:**
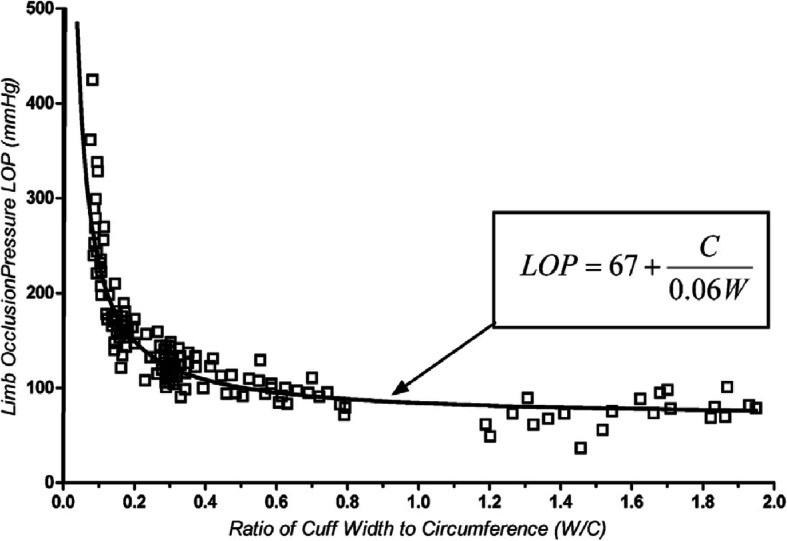
There is an inverse relationship between a patient’s limb occlusion pressure (LOP) vs the ratio of tourniquet cuff width to patient limb circumference. Thirty-four healthy normotensive volunteers were included in the study. The use of a wider tourniquet induces a lower LOP, and a lower tourniquet pressure level may be used to sufficiently occlude arterial blood flow into a limb. (Adapted with permission from Wolters Kluwer: Noordin S, McEwen JA, Kragh CJF, Eisen A, Masri BA. Surgical Tourniquets in Orthopaedics: The Journal of Bone and Joint Surgery-American Volume. 2009;91(12):2958–2967. Reprinted with permission of Wolters Kluwer from: Graham B, Breault MJ, McEwen JA, McGraw RW. Occlusion of Arterial Flow in the Extremities at Subsystolic Pressures Through the Use of Wide Tourniquet Cuffs: Clinical Orthopaedics and Related Research. 1993;(286):257–261. The Creative Commons license does not apply to this content. Use of the material in any format is prohibited without written permission from the publisher, Wolters Kluwer Health, Inc. Please contact permissions@lww.com for further information)

Tourniquet cuff design has a substantial impact on the pressure required to occlude blood flow past the cuff and the pressure gradient applied to the underlying tissue [1, 3, 6, 8, 9, 29]. Studies have demonstrated that underlying soft-tissue pressure and subcutaneous pressure at the cuff-limb interface decreases progressively as it nears the edges of a cuff [27, 28]. Also there is an inverse relationship between limb occlusion pressure and the ratio of cuff width to limb circumference, translating to high pressures required to achieve arterial occlusion with narrow cuffs (Fig. 2) [9]. There is a significantly positive relationship between cuff inflation pressure levels and the pressure gradient applied to the underlying tissue, and the difference between the pressure level seen at the cuff midpoint compared to the edges increases with higher set pressure levels [8, 9].

The bladder width, shape, and fit to the limb impact the distance over which the pressure changes between compressed to uncompressed tissue [8, 29]. A wide cuff ensures a greater distance for the applied pressure to change between the midpoint and edges of the cuff, reducing the applied pressure gradient [1, 3, 9, 24, 29]. Contoured tourniquet cuffs enable a better fit on tapered limbs; the transmission of inflation pressure to the underlying tissue is more effective [8]. Younger et al. compared final cuff pressure levels as a function of LOP between a wide contoured cuff and a standard cuff and found the mean cuff pressure level was reduced by 40 mmHg in the contoured cuff [29]. The adoption of wide cuffs that properly fit various limb shapes enables the use of lower pressure levels and pressure gradients, reducing the potential for nerve compression injury [29].

### Additional applications of tourniquets

Tourniquet usage has primarily focused on surgical applications. However, recent advances in tourniquet technology have included adaptations of surgical tourniquet designs for two new applications and settings: 1) pre-hospital tourniquets, and 2) blood flow restriction therapy. For these new applications and settings, safe tourniquet use beyond surgery remains informed by evidence arising from surgical tourniquet development, research, evaluation and clinical studies.

#### Pre-hospital tourniquets

**Fig. 3:**
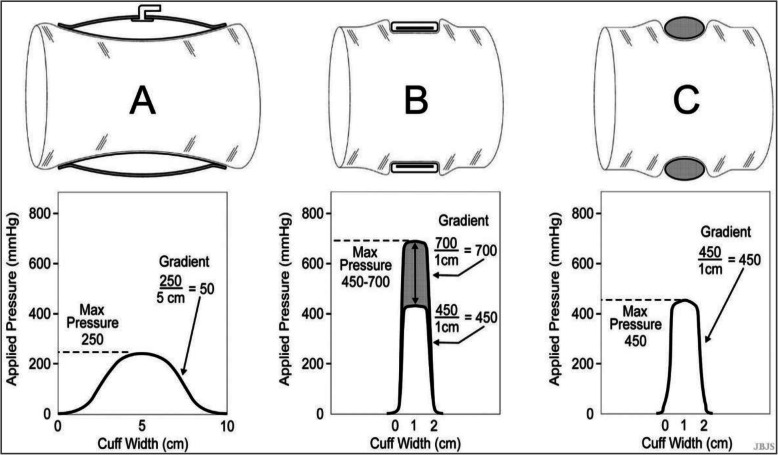
A comparison of the required pressure levels and applied pressure gradients for three types of cuff design. **a**: a wide, modern pneumatic surgical tourniquet cuff; **b**: a non-pneumatic, inelastic, belt-tightened cuff typically found in pre-hospital trauma settings; and **c**: a non-pneumatic elastic ring designed to be rolled up the limb to exsanguinate and occlude blood flow in one motion. The narrow designs of (**b**) and (**c**) require increased tourniquet pressure levels to occlude blood flow. The narrow distance for the changes in applied pressure to occur results in high applied pressure gradients in cuffs (**b**) and (**c**). (Reproduced with permission of Wolters Kluwer Health, Inc., from: Noordin S, McEwen JA, Kragh CJF, Eisen A, Masri BA. Surgical Tourniquets in Orthopaedics: The Journal of Bone and Joint Surgery-American Volume. 2009;91(12):2958–2967. The Creative Commons license does not apply to this content. Use of the material in any format is prohibited without written permission from the publisher, Wolters Kluwer Health, Inc. Please contact permissions@lww.com for further information)

Pre-hospital tourniquets are designed for short-term use to prevent traumatic blood loss and death by exsanguination, prior to admission to a hospital. Pre-hospital tourniquets are used in military applications, as well as emergency civilian situations. As a result, unlike the robust automatic pneumatic surgical tourniquet, pre-hospital tourniquets must necessarily be designed to be small and light-weight, but effective and suitable for use with minimal training [3, 11, 30]. These devices have typically been mechanical in nature. There have been significant improvements in the design of pre-hospital tourniquets for use in civilian settings [11, 30, 31]. However, they do not automatically monitor tourniquet application times, and many do not control and minimize tourniquet pressure levels and applied pressure gradients [30, 31]. It is not possible to actively regulate and maintain the level of applied pressure with such tourniquets, and the required pressure levels to occlude blood flow is substantially higher than used in automated pneumatic counterparts [30, 31]. The applied pressure gradients are also exceedingly high, increasing the probability of nerve injury (Fig. 3) [6, 8, 9].

There are currently no established guidelines for the evaluation and ongoing tourniquet management of patients with pre-applied tourniquets upon admission to the hospital. The differences in the pre-hospital tourniquet design mean that patients may be more at risk for tourniquet-related injury [11, 32]. For example, the objective should be to replace the pre-applied tourniquet with a safer, pneumatic hospital tourniquet instrument and cuff as soon as practical. It should be determined when the pre-hospital tourniquet was applied (duration), by whom, with what applied pressure, directly to skin or not (are there objects underneath?), and the type of device [11, 31, 32]. This information will allow the surgeon to better understand the extent of possible injuries, and to adapt their treatment plan to reduce any further risk of injury. There is a need for pre-hospital to hospital tourniquet transfer guidelines to be established, and a surgeon’s knowledge of surgical tourniquet safety may be useful in developing such recommendations.

#### Blood flow restriction (BFR)

Interest in BFR therapy has dramatically increased over the past 2 years, as clinical studies demonstrate the ability for this technique to enhance and accelerate patient recovery after surgery or injury [10]. BFR therapies use tourniquets to restrict arterial blood flow into limbs during low-load rehabilitative exercise, with the purpose of stimulating physiological responses that reduce muscle atrophy and increase muscle strength [10, 33, 34]. It has been proposed that BFR therapy is a particularly effective treatment method for joint-related procedures, including both the hip and the knee [35–38]. Preoperative BFR exercise improves muscle strength and reduces joint pain, and postoperative BFR exercise has a significant impact on functional improvement and recovery time by increasing hip and quadricep strength with low-impact exercises [10, 34–37]. A recent study has demonstrated that BFR may impact regenerative bone growth [37]. Early integration of BFR into the surgical treatment plan, and collaboration between the surgeon, physical therapist and patient may further improve functional improvement and recovery time. The safety and effectiveness of currently available equipment for BFR therapy differs widely [10, 39, 40]. Clinical studies generally conclude that BFR therapy can be provided safely and effectively to most patient populations using surgical-grade tourniquet technology, provided that the equipment is set by trained professionals with knowledge of appropriate protocols including personalized restriction pressure levels, application methods, duration limits, and the implications of different cuff designs and widths [10, 33, 39]. An integrated relationship between the surgeon and the physiotherapist in the prescription and administration of the therapy would both improve patient recovery and streamline the treatment plan. The advancements of safety learned through centuries of evolution of the surgical tourniquet would be carried forward to BFR and other perioperative applications to create safe and effective patient treatment protocols, and improve patient recovery.

## Conclusions

The modern pneumatic tourniquet is used extensively in surgical procedures, and additional applications of the tourniquet are emerging in pre-hospital, perioperative settings, rehabilitative medicine and physical training in athletes and the aged. Clinical studies over the past 50 years and more have demonstrated that the primary risk for tourniquet-related nerve injury is mechanical compression arising from high pressure levels in tourniquet cuffs and high applied pressure gradients near tourniquet cuff edges. Increasing age, obesity, and the presence of an underlying peripheral neuropathy are potential risk factors for tourniquet-induced nerve damage.

The advancements made in automatic, pneumatic tourniquet technology have significantly reduced the occurrence of tourniquet-related injuries, with implementation of lower, controlled, and personalized pressure levels and reduced applied pressure gradients. As new applications for tourniquet technology in the perioperative field emerge, it is essential that studies continue to reference and learn from the advancements made in surgical tourniquet technology (Table 1). The reduction of tourniquet pressure levels and applied pressure gradients decreases the risk of tourniquet-related injuries. Personalization of tourniquet pressure levels, the use of wide tourniquet cuffs, appropriate fit and application of the tourniquet cuff have all been shown in clinical studies to reduce pressure levels and applied pressure gradients. However, application of these methods and equipment has not yet become widely adopted in clinical practice. The next advancement in tourniquet safety will come from the revision and adoption of standards of practice for tourniquet use not only in surgery, but also in related trauma and rehabilitation environments.

## Data Availability

Data sharing not applicable to this article as no datasets were generated or analysed during the current study.

## Abbreviations

BFR: Blood Flow Restriction
LOP: Limb Occlusion Pressure
